# Exploring Relative Preferences for HIV Service Features Using Discrete Choice Experiments: a Synthetic Review

**DOI:** 10.1007/s11904-020-00520-3

**Published:** 2020-08-28

**Authors:** I Eshun-Wilson, H-Y Kim, S Schwartz, M Conte, D V Glidden, E H Geng

**Affiliations:** 1 Division of Infectious Disease, School of Medicine, Washington University in St. Louis, Childrens Pl, St. Louis, MO 63110 USA; 2 Department of Population Health, New York University School of Medicine, New York, USA; 3 Department of Epidemiology, Johns Hopkins Bloomberg School of Public Health, Baltimore, MD USA; 4 Zucker School of Medicine at Hofstra/Northwell in Hempstead, New York, USA; 5 Department of Epidemiology, University of California, San Francisco, USA

**Keywords:** Preference, Discrete choice experiment, Review, HIV, Service delivery, Differentiated care

## Abstract

**Purpose of Review:**

Aligning HIV treatment services with patient preferences can promote long-term engagement. A rising number of studies solicit such preferences using discrete choice experiments, but have not been systematically reviewed to seek generalizable insights. Using a systematic search, we identified eleven choice experiments evaluating preferences for HIV treatment services published between 2004 and 2020.

**Recent Findings:**

Across settings, the strongest preference was for nice, patient-centered providers, for which participants were willing to trade considerable amounts of time, money, and travel distance. In low- and middle-income countries, participants also preferred collecting antiretroviral therapy (ART) less frequently than 1 monthly, but showed no strong preference for 3-compared with 6-month refill frequency. Facility waiting times and travel distances were also important but were frequently outranked by stronger preferences. Health facility–based services were preferred to community- or home-based services, but this preference varied by setting. In high-income countries, the availability of unscheduled appointments was highly valued. Stigma was rarely explored and costs were a ubiquitous driver of preferences.

**Summary:**

While present improvement efforts have focused on designs to enhance access (reduced waiting time, travel distance, and ART refill frequency), few initiatives focus on the patient-provider interaction, which represents a promising critical area for inquiry and investment. If HIV programs hope to truly deliver patient-centered care, they will need to incorporate patient preferences into service delivery strategies. Discrete choice experiments can not only inform such strategies but also contribute to prioritization efforts for policy-making decisions.

## Introduction

Improving engagement in HIV care will require an understanding of patient experiences and patient-centered approaches for service delivery [1–3]. Over the last two decades, a robust HIV response has had a substantial impact on increasing the number of people living with HIV (PLWH) who know their HIV status (79% in 2018) [4]. The comparative progress of engaging PLWH in long-term HIV treatment services has, however, lagged behind. High disengagement rates [5] and repeated transitions in and out of care even for those established on antiretroviral therapy (ART) [6–9] have resulted in an increased focus on identifying effective strategies to improve linkage and retention in HIV services [10].

Given the numerous options for how and where to make improvements to HIV services, identifying which service features are most important to PLWH can aid future intervention design and prioritization. Differentiated services delivery models which aim to deliver more patient-centered services by varying several aspects of service delivery in Sub-Saharan Africa, such as visit frequency, waiting time, and service location, have to date shown modest effects on treatment outcomes [2, 11], suggesting that more needs to be considered to improve engagement in HIV care. These models have been informed by the need to decongest health services and a wealth of research identifying barriers to HIV care including: competing work and family priorities, economic costs, stigma, overburdened and incoordinate health services, and disrespectful health workers [12–14]. Although these barriers to care are well articulated, it is unclear what their relative importance is with respect to one another. Determining how service delivery features are valued can enrich our understanding of how PLWH make engagement decisions and further inform the design of patient-centered services.

Discrete choice experiments (or conjoint analysis) are a survey tool commonly used in marketing research to identify relative preferences for the attributes (characteristics) of a service or product, now increasingly used in health to evaluate service delivery preferences to inform intervention design, implementation, and policy [15, 16]. Choice experiments can help identify what is important to PLWH, and additionally define how this varies for population sub-groups. Both main preferences and preference heterogeneity can inform what works, for who, where, and under what circumstances, to inform and optimally target and adapt implementation strategies.

Although preference estimates are fairly population and context specific, the recent proliferation of choice experiments evaluating HIV service delivery suggests that synthesizing data across contexts may help identify cross-cutting service features of importance to PLWH. We therefore synthesized evidence from choice experiments evaluating preferences for HIV services to determine which service features are of greatest importance and to provide insight into the trade-offs PLWH are willing to make to get the type of service they want.

## Methods

We identified choice experiments evaluating HIV service delivery published between 1 Jan 2004 and 30 Jan 2020 from searches conducted in PubMed, using terms for HIV or antiretroviral therapy combined with the following terms: DCE or “discrete choice” or conjoint or preference. We additionally searched conference abstracts from the Conference on Retroviruses and Opportunistic Infections (CROI), International AIDS Society conferences (IAS/AIDS), and ISPOR (International Society for Pharmacoeconomics and Outcomes Research), for the last 2 years.

We evaluated the methodological quality of the included studies using the ISPOR checklist [17]. This 10-item checklist presents the steps involved in conducting and reporting good discrete choice experiments, by evaluating the following: (1) the research question; (2) the attributes and levels; (3) the construction of tasks; (4) the experimental design; (5) the preference elicitation; (6) the instrument design; (7) the data collection plan; (8) the statistical analyses; (9) the results and conclusions; and (10) the study presentation.

We abstracted descriptive data regarding study questions, study methods, attributes, and attribute levels, and in order to compare relative preferences across experiments, we ranked the importance of each attribute within each experiment and then compared this ranking of attribute pairs across experiments.

For example, if two studies both included provider attitude and type of health facility, and in both studies provider attitude ranked higher (more important, e.g., ranked 1) than type of health facility (e.g., ranked 3), we captured this finding and presented it in both tabular and graphical formats. Attribute ranking within each experiment was based on the coefficients (i.e., utility or preference weight) estimated from the models. The relative importance of each attribute was measured as the difference in the model coefficients from the level with the maximum utility to the level with the minimum utility. We set the baseline category to the attribute level with the lowest utility, to standardize the measurement of the relative importance independent of the method of coding (dummy or effect coding) or the types of statistical models such as conditional logit or mixed logit regressions. If a study reported odds ratios (ORs) rather than the coefficients, we took the ratio of the ORs with the largest value to that with the lowest value (for example, for an attribute with an OR of 2.0 for one level and an OR of 0.5 for another level, the ratio would be 2.0/0.5 = 4.0). For continuous variables such as waiting time or cost, if the study reported coefficients on a continuous scale (i.e., coefficient per unit increase), we calculated the coefficients for the discrete levels as presented to participants in the choice experiment and evaluated the relative importance. For example, if the utility (coefficient) per hour of waiting time was − 0.175 and the levels presented in the experiment were 30 min, 2 h, and 5 h, if we set the default level at 30 min, then the 30 min coefficient would be (0.5) × (− 0.175) = − 0.09 and the relative utility for 5 h as compared with that for 30 min would be (5 − 0.5) × (− 0.175) = − 0.79.

Once the attributes were ranked in each study, we qualitatively generated overarching attribute categories based on similarities between the attributes presented in each experiment: for example, one study may have named an attribute “Attitude of staff at health facility” while others may have named similar attributes “Provider attitude” or “Patient-centered care”; we explored these and their levels and collectively named them “Provider attitude” for the purposes of comparison between experiments. We descriptively summarized and tabulated the ranking of all attributes according to these categories. Among the key attributes that were explored in more than three studies, we also generated bar charts (relative utility ranking plots) to represent the “higher” or “lower” ranking of each attribute relative to other attributes. These plots help visualize how frequently a particular attribute is preferred compared with other attributes presented in the choice experiments; each graph represents the relative ranking for a single attribute (e.g., provider attitude) compared with all other attributes presented in studies including that main attribute. The number of studies which evaluated an attribute pair (including the main attribute) is presented on the *x*-axis, and all comparison attributes are presented on the *y*-axis.

### Characteristics of Included Discrete Choice Experiments

Searches yielded 1226 records which after screening resulted in eight included studies and 11 choice experiments [18–25]. Study settings, populations, and methods are summarized in Table 1. Studies included participants from six low- and middle-income countries: two each from South Africa and Zambia and one each from Ethiopia, Mozambique, Kenya, and Zimbabwe. A further two studies from high-income countries contributed to the synthesis: one from the UK and another from the USA. Experiments were conducted in adults living with HIV in ten experiments, and among these, two were restricted to women; one was among patients lost to care; another was among those in unstable housing. One study evaluated preferences in community members of unknown HIV status. The majority of experiments explored general preferences for HIV services; three specifically explored preferences for differentiated service delivery, two evaluated preferences for appointments at an HIV clinic or a general practitioner, and another two experiments explored preferences for private pay for service HIV care. Samples ranged from 65 to 1013 participants.

**Table 1:** Characteristics of included studies

Paper	Study characteristics			Design features				Analytic methods	
	Country	Population	Sample size	Question	Number of attributes	Number of scenarios	Number of tasks*	Analytic method	Evaluation of trade-offs or marginal utilities
Kruk 2016a [18]	Ethiopia	HIV-positive adults: women	1013	Preferences for HIV services	6	2	9	Mixed logit	Willingness to trade
Kruk 2016b [18]	Mozambique	HIV-positive adults: women	1020	Preferences for HIV services	6	2	9	Mixed logit	Willingness to trade
Miners 2017a [19]	England, UK	HIV-positive adults	1106	Preferences for HIV clinic appointments	4	2	12	Conditional logit and latent class	Not reported
Miners 2017b [19]	England, UK	HIV-positive adults	1106	Preferences for general practitioner appointments	5	2	12	Conditional logit and latent class	Not reported
Opuni 2010a [20]	South Africa	HIV-positive adults	510	Preferences for private (fee for service) HIV clinics	4	3	20	Two-level random intercept logit	Probability of purchase
Opuni 2010b [20]	South Africa	HIV unknown community members	777	Preferences for private (fee for service) HIV clinics	4	3	20	Two-level random intercept logit	Probability of purchase
Zanolini 2018 [21]	Zambia	HIV-positive adults: lost to follow-up	280	Preferences for HIV services	5	2	9	Mixed logit	Willingness to trade
Conte 2020 [22]	USA	HIV-positive adults: unstable housing	65	Preferences for HIV services	5	2	12	Mixed logit	Willingness to trade
Eshun-Wilson 2019 [23]	Zambia	HIV-positive adults on ART	486	Preferences for HIV DSD**	6	2	7	Mixed logit	Willingness to trade
Rabkin 2020 [24]	Zimbabwe	HIV-positive adults on ART	500	Preferences for HIV DSD**	7	2	8	Fixed effects logit model	Not reported
Dommaraju 2020 [25]	Kenya	HIV-positive adults on ART	104	Preferences for HIV DSD**	7	3	10	Hierarchical Bayesian analysis	Not reported

*Per participant

**Differentiated service delivery

### Methodological Quality of Included Studies

Overall, after the application of the ISPOR checklist, we found that the methodological quality of the included studies was good overall, but varied across domains, with some domains consistently reported well and others poorly across studies.

All eight studies (presenting 11 DCEs) appeared to have well-defined research questions and chose attributes that were supported by evidence, including literature reviews and qualitative research. Choice tasks were constructed appropriately, with justification of the number of attributes and profiles in each conjoint task. Two studies included an opt-out/status-quo question, a practice which is discouraged due to potential impact on preference elicitation and estimations [21, 26]. The choice of experimental design was justified and evaluated in the majority, with studies specifying orthogonal or near-orthogonal designs. Two studies did not report on the properties of the experimental design in terms of efficiency score, correlation of attributes, balance, or overlap [19, 20], and one study presented 20 questions to each participant which was higher than the reported average of 8–16 questions and can result in response fatigue [20, 27]. There was little discussion of preference elicitation during the administration of the experiment, and methods to establish comprehension or the inclusion of qualifying questions were infrequent: with only one study including a follow-up qualifying question assessing whether participants would actually pay for the clinic they had chosen [20].

The majority of data collection instruments were piloted first and collected relevant demographic information on participants. Tools contained relatively clear explanations of the attributes and their levels. Data collection was similarly well described, and mode of administration of the experiment (cards, paper forms, electronic tools) was reported in all but one study [21]. Five studies either used the sample size estimation based on the formula *N* ≥ (500 × c)/(*a* × *t*) [28]—where *N* is the number of participants, *t* is the number of choice tasks, *a* is the number of alternative scenarios, and *c* is the largest number of attribute levels for any one attribute [23–26]—or followed the proposed rule of 300 or more participants and 200 per sub-group [21]. Three studies did not present sample size calculations [18–20], but these studies had samples of over 1000 participants which may have been sufficient to estimate effects and accommodate sub-grouping and interactions. Ethical considerations were reported in all studies.

Reporting of analytic methods was less clear: although the respondent characteristics were presented in the studies, these were infrequently compared with characteristics of the source population [29]; the quality of responses was also infrequently assessed, with only three studies exploring internal validity through the use of dominant and repeat questions [18, 21, 24]. Several studies included attributes which were modeled continuously, but tests to explore whether the data was consistent with linear, log, quadratic, or other functional forms were lacking. The specification of dummy or effects coding was only reported in two studies [19, 20]. Sub-group analyses and interactions were used to explore preference heterogeneity in nine studies, and ten studies used appropriate models to account for unobserved preference variation across respondents including mixed logit, latent class, two-level random intercept, and hierarchical Bayes models. All studies provided an interpretation of the relative value of specific attributes. Seven studies presented willingness to pay/trade estimates in the form of willingness to wait (the waiting time people were willing to tolerate [21, 23]), willingness to travel analysis [21], willingness to pay analysis, and probability of purchase which quantifies how much money people would trade for another appealing attribute or pay for a particular service [18, 20, 26]. The majority of studies described relevant study limitations, and conclusions and implications were appropriate.

### Results of Synthesis and Utility Ranking Across Discrete Choice Experiments

Twenty-four different attributes were examined across the experiments (Table 2). There was heterogeneity in the types of attributes and attribute levels explored, and variation in presented levels was more pronounced for some continuous variables (e.g., cost of services and refill frequency) than others (e.g., waiting time, distance, and opening hours). The most frequently included service attributes were provider attitude, cost of services, waiting time, and ART refill frequency.

**Table 2:**
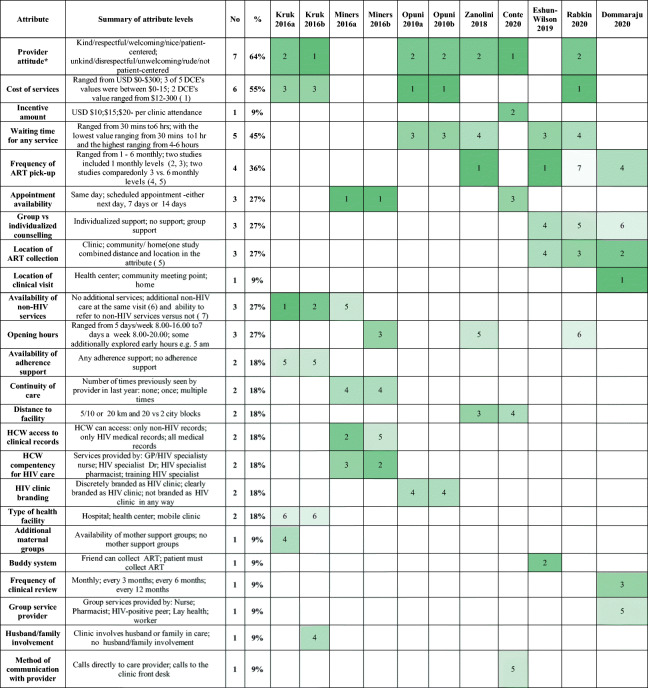
Summary of attribute levels and utility importance rankings within studies

Darker colors represent higher ranking and lighter colors represent lower ranking within individual DCEs. No: represents the number of experiments evaluating the attribute; % : represents the percentage of studies evaluating the attribute

*Most studies presented provider attitude as dichotomized, Opuni included a third level of indifferent providers

^1^Opuni M, Bishai D, Gray GE, McIntyre JA, Martinson NA. Preferences for characteristics of antiretroviral therapy provision in Johannesburg, South Africa: results of a conjoint analysis. *AIDS and behavior*. 2010;14 [4]:807–15

^2^Zanolini A, Sikombe K, Sikazwe I, Eshun-Wilson I, Somwe P, Bolton Moore C, et al. Understanding preferences for HIV care and treatment in Zambia: evidence from a discrete choice experiment among patients who have been lost to follow-up. *PLoS medicine*. 2018;15 [8]:e1002636

^3^Eshun-Wilson I, Mukumbwa-Mwenechanya M, Kim HY, Zannolini A, Mwamba CP, Dowdy D, et al. Differentiated care preferences of stable patients on antiretroviral therapy in Zambia: a discrete choice experiment. *Journal of acquired immune deficiency syndromes* (1999). 2019;81 [5]:540–6

^4^Dommaraju S, Hagey J, Odeny T, Okaka S, Kadima J, Bukusi EA, et al. Preferences of people living with HIV for differentiated care models in Kenya: a discrete choice experiment. In press Author contact: SagarDommaraju@northwesternedu 2020

^5^Rabkin M, Strauss M, Mantell JE, Mapingure M, Masvawure TB, Lamb MR, et al. Optimizing differentiated treatment models for people living with HIV in urban Zimbabwe: findings from a mixed methods study. PloS one. 2020;15 [1]:e0228148

^6^Kruk ME, Riley PL, Palma AM, Adhikari S, Ahoua L, Arnaldo C, et al. How can the health system retain women in HIV treatment for a lifetime? A discrete choice experiment in Ethiopia and Mozambique. PloS one. 2016;11 [8]:e0160764

^7^Miners AH, Llewellyn CD, Cooper VL, Youssef E, Pollard AJ, Lagarde M, et al. A discrete choice experiment to assess people living with HIV’s (PLWHIV’s) preferences for GP or HIV clinic appointments. *Sexually transmitted infections*. 2017;93 [2]:105–11

^8^ Conte M, Eshun-Wilson I, Geng E, Imbert E, Hickey MD, Havlir D, et al. Understanding preferences for HIV care amongst patients experiencing homelessness or unstable-housing: A discrete choice experiment. AIDS Virtual conference: July 7-10, 2020 Abstract number OAE01. 2020. [22]

#### Good Provider Attitude Is Highly Valued

**Fig. 1:**
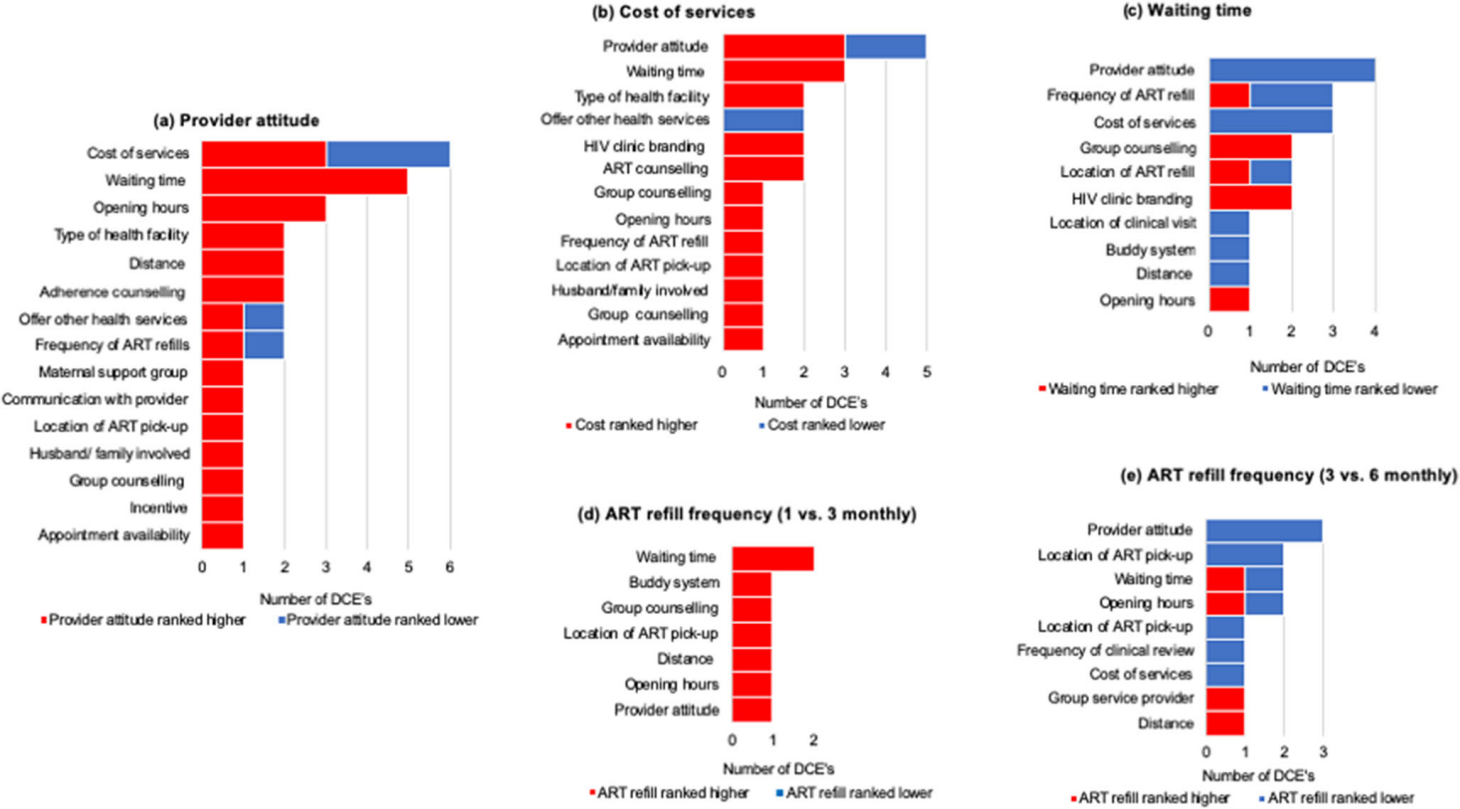
Relative preference rankings for the most commonly reported HIV service attributes: **a** provider attitude, **b** cost, and **c** waiting, **d** & **e** refill frequency. Length of the bar (*x*-axis) represents the number of studies with a direct head-to-head comparison of the attribute listed on the *y*-axis; the distribution of colors within the bar represents the studies for which the attribute of interest was ranked higher (dark green) or lower (light green) relative to the other attributes (on the *y*-axis).

Preference data from seven choice experiments that explored provider attitude revealed that participants almost always valued provider attitude above other service features, with provider attitude ranking consistently higher in 12 of 15 relative preference comparisons (Fig. 1a). Provider attitude was most frequently dichotomized and participants routinely preferred “nice” patient-centered, respectful providers as opposed to “rude,” not patient-centered or disrespectful providers. In one choice experiment from South Africa which additionally explored an intermediate group of “indifferent” providers, the relative utility for indifferent providers compared with that for kind providers was approximately half of that for rude providers compared with that for kind providers and was equivalent to the preference for facility waiting time of 5 h compared with 30 min, suggesting that if PLWH could have a 30-min waiting time at the clinic instead of 5 h of waiting time, they may be willing to accept an indifferent provider instead of a kind provider [20].

#### Service Costs Are a Major Concern, But Very Minimal Costs Are Acceptable to Most

Five choice experiments from three studies explored cost of services (Fig. 1b). In general, PLWH in low- and middle-income countries (LMICs) did not want to pay for services, and cost ranked higher than other service attributes in 11 of 13 attribute comparisons. However, this ranking was based on the preference for the highest compared with the lowest cost. When the presented cost values were explored, it appeared that small costs of approximately $1–$3 had comparable utility to free services and ranked lower than all other service attributes, suggesting that participants in Ethiopia, Mozambique, and Zimbabwe would be willing to pay these small amounts for services [18, 24]. In these settings, PLWH were also willing to accept costs of approximately $5–6 if they could have other strongly desired service features (such as nice providers) and in South Africa, PLWH had a 50% probability of selecting a service which cost approximately $5 at the time [18, 20]. Beyond this threshold, cost was less preferred and at values of $10–$12, cost became more important than several other service attributes.

#### Waiting Time of up to 6 h Is Less Important Than Other Strongly Preferred Service Features

Waiting time frequently ranked lower than other attributes (3rd or 4th) irrespective of the range of time presented to participants (Fig. 1c). The waiting times presented to participants across experiments were comparable, with the lowest level ranging from 30 min to 1 h in all experiments and the longest waiting time ranging from 4 to 6 h. Waiting time ranked lower than other preferences for provider attitude, cost, location of services, buddy ART pick-up systems, and distance to services. Waiting time was only consistently valued above other attributes in a few instances—when compared with counseling services, HIV clinic branding within the health facility, and opening hours.

#### PLWH Do Not Want to Receive 1 Monthly ART Refills and Appear to Prefer 3 or 6 Monthly Refills Equally

Evidence from four choice experiments exploring preferences for ART refill frequency indicated that participants did not want to receive monthly ART refills and preferred three to six monthly refills equally. Two choice experiments compared one versus three or five monthly refills [21, 23]—in these studies, refill frequency was ranked highest above all other attributes, with an overwhelming preference for not obtaining refills on a monthly basis (Fig. 1d); in the three choice experiments which evaluated three versus five or six monthly refills, ART refill frequency ranked lower than provider attitude in all, and additionally ranked lower than opening hours, waiting time, and group or individual counseling services in two (Fig. 1e) [24].

#### Clinic Opening Hours Are Frequently Seen as the Least Important Service Attribute

Three DCEs included attributes exploring preferences for clinic opening hours which included extending beyond regular opening hours into afternoons, evenings, weekends, or early mornings; overall, the ranking of relative utilities for this attribute within choice experiments was low—ranking 3rd [19] or 5th and 6th [21, 25].

#### PLWH Prefer to Collect ART at the Health Facility, But Overall, Service Location Is Less Important Than Other Key Service Attributes and Shows Marked Preference Heterogeneity

Location of services, evaluated in three experiments, including community- versus home- or clinic-based services, had variable rankings, with location ranked highest compared with other attributes within one experiment which did not include provider attitude or financial attributes but lower in other studies where it was displaced by provider attitude, cost, and short ART refills. In two experiments, PLWH preferred the health facility to community- or home-based services, but one experiment demonstrated marked preference heterogeneity, with rural participants showing a preference for community-based services and urban participants showing a preference for the health facility [23]. Two experiments additionally compared home to community services; one study showed no difference [24] and another stronger preferences for the community instead of home-based services [25].

#### Large Distances Between Home and the Health Facility Are Undesirable But Acceptable When Compared with Other Highly Valued Service Attributes

Distance to the facility was consistently outranked in two DCEs by cost or incentives, provider attitude, ART refill frequency (one vs three monthly), and appointment availability (irrespective of distance presented), but the furthest distance (20 km compared with 5 km) ranked higher than waiting time, opening hours, and traveling 20 blocks instead of 2 blocks ranked higher than the method of communication with providers.

#### Appointment Availability Is of Particular Importance in High-Income Settings

Appointment availability which included the ability to drop-in for unscheduled visits and same-day appointments was evaluated in three experiments from the USA and the UK—this ranked highly when compared with service features such as opening hours, continuity of care, healthcare worker (HCW) competency, and access to clinical records [19] but ranked lower in one experiment when compared with provider attitude and incentives [26].

#### Types of Counseling and Adherence Support Are of Very Low Importance Compared with Other Attributes

Preferences for group versus individualized counseling ranked generally lower than all other attributes presented in the three experiments considering this attribute. The availability of routine ART adherence support ranked poorly in the two experiments [18], surpassed by preferences for costs, provider attitude, availability of non-HIV services, partner/family involvement in care, and maternal support groups.

#### Provision of Integrated Care Is Important in Some Settings, and Health Worker Competency and Continuity of Care Have Low Relative Utility

The provision of additional non-HIV services at the same visit was highly valued in two experiments from one study [18] in Sub-Saharan Africa, but had low ranking in a UK-based study where health workers referred patients to additional services without actually providing the non-HIV service themselves [19]. The level of access HCWs had to clinical records and also the competency of HCWs was reviewed in the two choice experiments from the UK and had variable rankings—both ranking lower than appointment availability which was the most valued attribute in this setting. Continuity of care was similarly only evaluated in the two UK choice experiments and had a relatively low ranking (4th) in both choice experiments. HIV clinic branding which explored preferences for how evident it was that PLWH were attending HIV services to others at the health facility ranked poorly when compared with preferences for cost, provider attitude, and waiting time in two experiments. Further attributes which were only explored in a single study are presented in Table 2.

## Discussion

Despite substantial heterogeneity in experiments with regard to context, attributes, attribute levels, and relative utilities, this synthesis revealed a several key preferences which were identified across population groups. First, PLWH prefer having nice as opposed to rude providers, and for many, this is more important than the majority of other HIV service features which can be offered. Second, participants in LMICs prefer free services but would be willing to accept very minimal costs to get the services they desire; as costs rise, however, cost of services rapidly becomes more important than all other service features. Third, when presented with a range of DSD service features, participants in LMICs prefer three or six monthly ART refills to one monthly refills. And although PLWH do not want to travel large distances or wait for long periods at the health facility, when compared with other desirable service features such as nice providers, low-cost services, and infrequent clinic visits, these preferences are of less importance. Fourth, the majority of PLWH in LMICs prefer getting ART at a health facility rather than in the community or at home. And last, in high-income countries, specifically, PLWH highly value the ability to have unscheduled “drop-in” HIV care as compared with scheduled clinic appointments.

The strong preference for “nice” providers demonstrated in this synthesis is supported by a wealth of qualitative research exploring patient experiences with HIV services [12, 14, 30, 31]. PLWH lost to services in Zambia, Tanzania, and Kenya reported negative provider attitude—including disrespect, humiliation, and punishment—as a common factor contributing to disengagement and re-engagement decisions [31]. In one choice experiment from the USA, the strongest preference was for providers “that know me as a person” compared with “providers who don’t know me as person,” highlighting the importance of the quality of the patient-provider relationship in this setting [22]. In LMICs, the preference for nice providers relative to other service delivery features may to some degree reflect the dichotomized nature in which provider attitude was presented to participants: in reality, the quality of patient-provider relationships is likely to follow a continuum, where some providers are “rude” and some “nice” and some falling somewhere in-between. One choice experiment from South Africa explored this intermediate group and found that PLWH preferred a nice provider to a rude provider and this utility was greater than any preference for waiting time, but the preference for a nice provider versus an indifferent provider was equivalent to the preference for waiting for 30 min instead of 5 h—in other words, patients may be willing to accept an indifferent provider if they could have a 30-min instead of a 5-h waiting time, indicating that patients may be willing to make some trade-offs with provider attitude [20]. Future choice experiments should consider including more moderate gradations of provider attitude to allow for further exploration of relative preferences and trade-offs for other service attributes. This should however not detract from the fact that provider attitude was the most important attribute driving patient care preferences: strategies to improve patient-provider relationships are essential if programs aim to maintain long-term engagement in HIV care [10].

It is unsurprising that cost of services was one of the attributes with the strongest preference utilities relative to several other service features. PLWH in LMICs were willing to accept only the most minimal costs, beyond which this service feature became increasingly undesirable. Although HIV services are currently free in most settings, shifts in donor funding and large unsustainable national HIV budgets are driving considerations of other models of care which incorporate public-private partnerships and co-payments for PLWH [20, 32]. Results from these experiments indicate that beyond the smallest costs, paying for services could have a substantial impact on engagement in HIV care.

Current international guidelines recommend reduced ART refill frequency to at minimum two to three monthly intervals to decongest HIV services and reduce structural obstacles for PLWH [33, 34]. Evidence from two choice experiments in this synthesis showed a strong relative preference for three monthly as opposed to one monthly refills compared with other service attributes, and a further three experiments demonstrate no differences in preference for longer refill intervals when compared with 3-month prescribing. This mirrors findings from observational studies and trials which demonstrate improved retention with three or six monthly versus one monthly ART refill frequency [33, 35] but minimal difference in HIV treatment outcomes for three versus six monthly refill comparisons [36, 37]. Efforts should be made to optimize drug supply chains and pharmacy storage capacity to ensure that all settings are able to provide at minimum 3-month refills and where possible longer drug supplies should be considered [38].

Other preferences for service features such as distance, waiting time, and location of services—prominent features adapted by DSD models—showed modest preferences when compared with other desirable service features, such as low costs and nice providers. The strength of preferences for distance and waiting time appeared similar across experiments—participants did not want to wait for too long or travel too far. The location of ART services however showed more heterogeneity; overall, PLWH preferred getting HIV care at health facility rather than in the community, but there was substantial variation within and across experiments, demonstrating that this particular service feature is highly sensitive to context.

Context also influenced the attributes presented in choice experiments: in high-income countries, the availability of unscheduled appointments was an important service attribute which was highly valued by PLWH and attributes related to the provider characteristics and patient-provider relationships were also presented more frequently in this setting than in LMICs. Features of DSD models such as refill frequency and location of services were exclusively presented in LMICs.

The role of stigma in HIV services was minimally explored in all choice experiments. Stigma concerns have been linked to which HIV services people choose and why they disengage [39–41], exemplified by a recent report from South Africa where stigma substantially reduced the desirability of community-based adherence clubs compared with facility-based services [40]. Stigma was included in the form of HIV clinic branding in one choice experiment and in this study ranked lower than all other attributes [20]. Stigma may have played a role in the preference for facility-based rather than community-based care in several experiments, but this was not explicit. A broader exploration of the relative importance of exposure and inadvertent HIV disclosure at clinics and community-based services can help inform future intervention design.

Overall, the choice experiments were of good quality, but two methodological elements were consistently under-reported—preference elicitation and statistical methods. Descriptions of preference elicitation were superficial—in addition to a general explanation of the choice experiment to participants, additional test questions, best-worst scaling, or confidence ratings can help support selected choices and confirm participants’ comprehension of the experiment [17, 42]. Statistical methods related to design and analysis were in many cases insufficiently detailed with few studies presenting data to support internal or external validity, or model estimation approaches [17]. Internal validity can be assessed through the review of responses to a dominant question or exploration of results, and external validity through the comparison of respondents’ characteristics to those of the inference population or through stated and revealed preferences [29, 43]. Descriptions of model estimation should include a description of the coding mechanism, and if attributes are modeled continuously, the linear assumption should be examined. Dummy and effects coding are functionally equivalent, but the specification of the coding scheme can help with the interpretation of the utilities and constant term, and in some cases, effects coding may be the more appropriate approach [44, 45].

It must be noted that discrete choice experiments present hypothetical situations to participants, at times for service features which they never experienced and that the examination of such relative preferences may not fully reflect how choices are made in real life. In this synthesis, we tried to contextualize the findings within relevant qualitative literature and trial results; there was however a lack of head-to-head comparisons of DSD models with which to compare our findings [38]. Also the importance and rankings generated are only relevant to the comparisons made within an individual experiment.

## Conclusions

The findings from this synthesis have important implications: HIV programs need to incorporate strategies to improve provider attitude and patient-provider relationships—even in overburdened settings, supply chains need to be optimized to provide longer ART refills in LMICS and appointment scheduling systems in HIC settings need to incorporate greater flexibility. Our synthesis further highlights how discrete choice experiments can provide an additional research tool for exploring patient preferences for care, which can inform how HIV services need to adapt to better align with what PLWH want.

## References

[1] Duncombe C, Rosenblum S, Hellmann N, Holmes C, Wilkinson L, Biot M (2015). Reframing HIV care: putting people at the centre of antiretroviral delivery. Tropical medicine & international health: TM & IH.

[2] IAS. Differentiated Service Delivery 2018 [Available from: http://www.differentiatedservicedelivery.org.

[3] Geng EH, Holmes CB, Moshabela M, Sikazwe I, Petersen ML (2019). Personalized public health: an implementation research agenda for the HIV response and beyond. PLoS Med.

[4] UNAIDS. Global HIV & AIDS statistics 2019 [Available from: https://www.unaids.org/en/resources/fact-sheet.

[5] Sikazwe I, Eshun-Wilson I, Sikombe K, Czaicki N, Somwe P, Mody A (2019). Retention and viral suppression in a cohort of HIV patients on antiretroviral therapy in Zambia: regionally representative estimates using a multistage-sampling-based approach. PLoS Med.

[6] Mody A, Eshun-Wilson I, Sikombe K, Schwartz SR, Beres LK, Simbeza S (2019). Longitudinal engagement trajectories and risk of death among new ART starters in Zambia: a group-based multi-trajectory analysis. PLoS Med.

[7] Kaplan SR, Oosthuizen C, Stinson K, Little F, Euvrard J, Schomaker M (2017). Contemporary disengagement from antiretroviral therapy in Khayelitsha, South Africa: a cohort study. PLoS Med.

[8] Lee H, Hogan JW, Genberg BL, Wu XK, Musick BS, Mwangi A (2018). A state transition framework for patient-level modeling of engagement and retention in HIV care using longitudinal cohort data. Stat Med.

[9] Lee H, Wu XK, Genberg BL, Mugavero MJ, Cole SR, Lau B, et al. Beyond binary retention in HIV care: predictors of the dynamic processes of patient engagement, disengagement, and re-entry into care in a US clinical cohort. AIDS (London, England). 2018. 10.1097/QAD.0000000000001936 PMC6136972 30005018

[10] Ford N, Geng E, Ellman T, Orrell C, Ehrenkranz P, Sikazwe I (2020). Emerging priorities for HIV service delivery. PLoS Med.

[11] Long L, Kuchukhidze S, Pascoe S, Nichols B, Cele R, Govathson C, et al. Differentiated service delivery models for antiretroviral treatment of HIV in sub-Saharan Africa: a rapid systematic review. AMBIT Project Report Number 04.: Boston University and HE2RO; 2020.

[12] Ahmed S, Autrey J, Katz IT, Fox MP, Rosen S, Onoya D (2018). Why do people living with HIV not initiate treatment? A systematic review of qualitative evidence from low- and middle-income countries. Social science & medicine (1982).

[13] Geng EH, Odeny TA, Lyamuya R, Nakiwogga-Muwanga A, Diero L, Bwana M (2016). Retention in care and patient-reported reasons for undocumented transfer or stopping care among HIV-infected patients on antiretroviral therapy in Eastern Africa: application of a sampling-based approach. Clinical infectious diseases: an official publication of the Infectious Diseases Society of America.

[14] Eshun-Wilson I, Rohwer A, Hendricks L, Oliver S, Garner P (2019). Being HIV positive and staying on antiretroviral therapy in Africa: a qualitative systematic review and theoretical model. PLoS One.

[15] Humphrey JM, Naanyu V, MacDonald KR, Wools-Kaloustian K, Zimet GD (2019). Stated-preference research in HIV: a scoping review. PLoS One.

[16] Terris-Prestholt F, Neke N, Grund JM, Plotkin M, Kuringe E, Osaki H (2019). Using discrete choice experiments to inform the design of complex interventions. Trials..

[17] Bridges JF, Hauber AB, Marshall D, Lloyd A, Prosser LA, Regier DA (2011). Conjoint analysis applications in health--a checklist: a report of the ISPOR Good Research Practices for Conjoint Analysis Task Force. Value in health: the journal of the International Society for Pharmacoeconomics and Outcomes Research..

[18] Kruk ME, Riley PL, Palma AM, Adhikari S, Ahoua L, Arnaldo C (2016). How can the health system retain women in HIV treatment for a lifetime? A discrete choice experiment in Ethiopia and Mozambique. PLoS One.

[19] Miners AH, Llewellyn CD, Cooper VL, Youssef E, Pollard AJ, Lagarde M (2017). A discrete choice experiment to assess people living with HIV’s (PLWHIV’s) preferences for GP or HIV clinic appointments. Sex Transm Infect.

[20] Opuni M, Bishai D, Gray GE, McIntyre JA, Martinson NA (2010). Preferences for characteristics of antiretroviral therapy provision in Johannesburg, South Africa: results of a conjoint analysis. AIDS Behav.

[21] Zanolini A, Sikombe K, Sikazwe I, Eshun-Wilson I, Somwe P, Bolton Moore C (2018). Understanding preferences for HIV care and treatment in Zambia: evidence from a discrete choice experiment among patients who have been lost to follow-up. PLoS Med.

[22] Conte M, Eshun-Wilson I, Geng E, Imbert E, Hickey MD, Havlir D, et al. Understanding preferences for HIV care amongst patients experiencing homelessness or unstable-housing: A discrete choice experiment. AIDS Virtual conference: July 7-10, 2020 Abstract number OAE01. 2020. 10.1097/QAI.0000000000002476 PMC8028840 33136742

[23] Eshun-Wilson I, Mukumbwa-Mwenechanya M, Kim HY, Zannolini A, Mwamba CP, Dowdy D, et al. Differentiated care preferences of stable patients on antiretroviral therapy in Zambia: a discrete choice experiment. Journal of acquired immune deficiency syndromes (1999). 2019;81(5):540–6. 10.1097/QAI.0000000000002070 PMC6625870 31021988

[24] Rabkin M, Strauss M, Mantell JE, Mapingure M, Masvawure TB, Lamb MR (2020). Optimizing differentiated treatment models for people living with HIV in urban Zimbabwe: findings from a mixed methods study. PLoS One.

[25] Dommaraju S, Hagey J, Odeny T, Okaka S, Kadima J, Bukusi EA, et al. Preferences of people living with HIV for differentiated care models in Kenya: a discrete choice experiment. Author manuscript - in peer review: SagarDommaraju@northwesternedu 2020. 10.1371/journal.pone.0255650 PMC8386850 34432795

[26] Conte M, Eshun-Wilson I, Geng E, Imbert E, Hickey MD, Havlir D, et al. Understanding preferences for HIV care amongst patients experiencing homelessness or unstable-housing: a discrete choice experiment. AIDS Virtual conference: July 7-10, 2020. Abstract number OAE01. 10.1097/QAI.0000000000002476 PMC8028840 33136742

[27] Reed Johnson F, Lancsar E, Marshall D, Kilambi V, Muhlbacher A, Regier DA (2013). Constructing experimental designs for discrete-choice experiments: report of the ISPOR Conjoint Analysis Experimental Design Good Research Practices Task Force. Value in health: the journal of the International Society for Pharmacoeconomics and Outcomes Research..

[28] Orme B (2010). Getting started with conjoint analysis: strategies for product design and pricing research. Second Edition ed.

[29] Janssen EM, Marshall DA, Hauber AB, Bridges JFP (2017). Improving the quality of discrete-choice experiments in health: how can we assess validity and reliability?. Expert review of pharmacoeconomics & outcomes research.

[30] Campbell C, Scott K, Skovdal M, Madanhire C, Nyamukapa C, Gregson S (2015). A good patient? How notions of ‘a good patient’ affect patient-nurse relationships and ART adherence in Zimbabwe. BMC Infect Dis.

[31] Ware NC, Wyatt MA, Geng EH, Kaaya SF, Agbaji OO, Muyindike WR (2013). Toward an understanding of disengagement from HIV treatment and care in sub-Saharan Africa: a qualitative study. PLoS Med.

[32] Sulzbach S, De S, Wang W. The private sector role in HIV/AIDS in the context of an expanded global response: expenditure trends in five sub-Saharan African countries. Health Policy Plan. 2011:i72–84. 10.1093/heapol/czr031 21729920

[33] Mutasa-Apollo T, Ford N, Wiens M, Socias ME, Negussie E, Wu P, et al. Effect of frequency of clinic visits and medication pick-up on antiretroviral treatment outcomes: a systematic literature review and meta-analysis. J Int AIDS Soc. 2017;20. 10.7448/IAS.20.5.21647 PMC6192466 28770599

[34] WHO. What’s new in service delivery 2015 [Available from: http://apps.who.int/iris/bitstream/handle/10665/204461/WHO_HIV_2015.46_eng.pdf;jsessionid=CC08AD47F5EB88823EB70ADF68C68C1A?sequence=1.

[35] Mody A, Roy M, Sikombe K, Savory T, Holmes C, Bolton-Moore C (2018). Improved retention with 6-month clinic return intervals for stable human immunodeficiency virus-infected patients in Zambia. Clinical infectious diseases: an official publication of the Infectious Diseases Society of America..

[36] Lebelo K, Cassidy T, Grimsrud A, Keene C, Ndlovu S, Hayes H, et al. Twelve-month retention and viral load outcomes from a noninferiority cluster randomized trial extending adherence club ART refill dispensing intervals to 6-monthly. 10TH IAS CONFERENCE ON HIV SCIENCE; 21–24 July 2019; Mexico City2019.

[37] Fatti G, Ngorima-Mabhena N, Mothibi E, Muzenda T, Choto R, Kasu T, et al. Outcomes of three versus six-monthly dispensing of antiretroviral treatment (art) for stable HIV patients in community ART refill groups: a cluster-randomized trial in Zimbabwe. J Acquir Immune Defic Syndr. 1999;2020. 10.1097/QAI.0000000000002333 PMC7172979 32097252

[38] Roy M, Bolton Moore C, Sikazwe I, Holmes CB (2019). A review of differentiated service delivery for HIV treatment: effectiveness, mechanisms, targeting, and scale. Current HIV/AIDS reports.

[39] Adherence, linkage and retention-in-care in antiretroviral treatment programmes in low and middle income countries: systematic review and synthesis of qualitative research [Internet]. 2017. Available from: http://www.crd.york.ac.uk/PROSPERO/display_record.php?ID=CRD42017057335.

[40] Mudavanhu M, West NS, Schwartz SR, Mutunga L, Keyser V, Bassett J, et al. Perceptions of community and clinic-based adherence clubs for patients stable on antiretroviral treatment: a mixed methods study. AIDS Behav. 2019. 10.1007/s10461-019-02681-8 PMC9933863 31560093

[41] Nyblade L, Stockton MA, Giger K, Bond V, Ekstrand ML, Lean RM (2019). Stigma in health facilities: why it matters and how we can change it. BMC Med.

[42] Muhlbacher AC, Kaczynski A, Zweifel P, Johnson FR (2016). Experimental measurement of preferences in health and healthcare using best-worst scaling: an overview. Heal Econ Rev.

[43] Strauss M, George G, Mantell JE, Romo ML, Mwai E, Nyaga EN (2018). Stated and revealed preferences for HIV testing: can oral self-testing help to increase uptake amongst truck drivers in Kenya?. BMC Public Health.

[44] Bech M, Gyrd-Hansen D (2005). Effects coding in discrete choice experiments. Health Econ.

[45] Hauber AB, Gonzalez JM, Groothuis-Oudshoorn CG, Prior T, Marshall DA, Cunningham C (2016). Statistical methods for the analysis of discrete choice experiments: a report of the ISPOR Conjoint Analysis Good Research Practices Task Force. Value in health: the journal of the International Society for Pharmacoeconomics and Outcomes Research.

